# In Silico Design of Potential Small-Molecule Antibiotic Adjuvants against *Salmonella typhimurium* Ortho Acetyl Sulphydrylase Synthase to Address Antimicrobial Resistance

**DOI:** 10.3390/ph17050543

**Published:** 2024-04-23

**Authors:** Oluwadunni F. Elebiju, Gbolahan O. Oduselu, Temitope A. Ogunnupebi, Olayinka O. Ajani, Ezekiel Adebiyi

**Affiliations:** 1 Department of Chemistry, College of Science and Technology, Covenant University Bioinformatics Research (CUBRe), Covenant University, Ota 112233, Ogun State, Nigeria; oluwadunni.elebijupgs@stu.cu.edu.ng (O.F.E.); gbolahan.oduselu@covenantuniversity.edu.ng (G.O.O.); temitope.ogunnupebipgs@stu.cu.edu.ng (T.A.O.); ola.ajani@covenantuniversity.edu.ng (O.O.A.); 2 Department of Chemistry, College of Science and Technology, Covenant University, Ota 112233, Ogun State, Nigeria; 3 Division of Applied Bioinformatics, German Cancer Research Center (DKFZ), 69120 Heidelberg, Germany

**Keywords:** adjuvants, antibiotics, cysteine biosynthesis, *St*OASS, drug resistance, scaffold hopping, toxicity

## Abstract

The inhibition of *O*-acetyl sulphydrylase synthase isoforms has been reported to represent a promising approach for the development of antibiotic adjuvants. This occurs via the organism developing an unpaired oxidative stress response, causing a reduction in antibiotic resistance in vegetative and swarm cell populations. This consequently increases the effectiveness of conventional antibiotics at lower doses. This study aimed to predict potential inhibitors of *Salmonella typhimurium* ortho acetyl sulphydrylase synthase (*St*OASS), which has lower binding energy than the cocrystalized ligand pyridoxal 5 phosphate (PLP), using a computer-aided drug design approach including pharmacophore modeling, virtual screening, and in silico ADMET (Absorption, Distribution, Metabolism, Excretion, and Toxicity) evaluation. The screening and molecular docking of 4254 compounds obtained from the PubChem database were carried out using AutoDock vina, while a post-screening analysis was carried out using Discovery Studio. The best three hits were compounds with the PubChem IDs 118614633, 135715279, and 155773276, possessing binding affinities of −9.1, −8.9, and −8.8 kcal/mol, respectively. The in silico ADMET prediction showed that the pharmacokinetic properties of the best hits were relatively good. The optimization of the best three hits via scaffold hopping gave rise to 187 compounds, and they were docked against *St*OASS; this revealed that lead compound **1** had the lowest binding energy (−9.3 kcal/mol) and performed better than its parent compound 155773276. Lead compound **1**, with the best binding affinity, has a hydroxyl group in its structure and a change in the core heterocycle of its parent compound to benzimidazole, and pyrimidine introduces a synergistic effect and consequently increases the binding energy. The stability of the best hit and optimized compound at the *St*OASS active site was determined using RMSD, RMSF, radius of gyration, and SASA plots generated from a molecular dynamics simulation. The MD simulation results were also used to monitor how the introduction of new functional groups of optimized compounds contributes to the stability of ligands at the target active site. The improved binding affinity of these compounds compared to PLP and their toxicity profile, which is predicted to be mild, highlights them as good inhibitors of *St*OASS, and hence, possible antimicrobial adjuvants.

## 1. Introduction

The capacity of microorganisms to inhibit the effects of antimicrobial agents is known as antibiotic resistance. This phenomenon can be due to a depletion in the efficiency of antibiotics to stop growth in the bacterial population [1]. Antibiotic resistance is regarded as a threat to the global public health system, with some of its consequences ranging from prolonged admission in hospitals to increasing mortality rates due to treatment failure [2]. These consequences have a direct impact on patients, healthcare, and the economy, as a loss in productivity leads to an elevation in the poverty rate [2]. One of the action plans stated by the World Health Organization (WHO) in response to the request by the World Health Assembly (WHA) to proffer solutions to antibiotic resistance is the optimization of the use of antimicrobial medicines in human and animal health [3]. Resistance to antimicrobial agents is developed based on factors such as antibiotic misuse, inaccurate diagnosis, a delay in accurate diagnosis, etc. Some of these factors can be addressed via appropriate antibiotic stewardship [4]. Antimicrobial resistance reduces the number of effective antimicrobials available against different microbial infections; hence, there is a need to design and develop new antimicrobial agents [5]. An approach that is being used to develop new antimicrobial agents that address resistance is the investigation of non-essential targets, the study of the biochemical pathways they are linked to, and the development of inhibitors against these targets [6]. The inhibition of these non-essential pathways with small molecules has resulted in promising successes to reduce future resistance resurgence, with some of these molecules used in combination with antimicrobial agents and known as adjuvants [4].

The cysteine biosynthetic pathway is a non-essential target that has been reported to be productive in antibacterial therapy [7]. Biotin, coenzyme A, Fe−S clusters, glutathione, methionine, and penicillin are some of the sulfur-containing biomolecules with cysteine as their precursor [8]. The cysteine biosynthetic pathway is absent in mammals and present in bacteria and plants [9]. The last step of cysteine biosynthesis is catalyzed by serine acetyltransferase (SAT) and *O*-acetylserine sulfhydrylase (OASS) in enteric bacteria. A study on the cysteine biosynthetic pathway in *Salmonella typhimurium* revealed that inactivating this pathway can reduce vegetative and swarm cell populations’ antibiotic resistance through an unpaired oxidative stress response. This, in turn, results in conventional antibiotics (e.g., Triazole) becoming effective at lower doses [10]. This highlights the possibility that treatment efficacy can be enhanced using cysteine biosynthesis inhibitors by reducing the antibiotic dosage, consequently decreasing resistance and its spread [7]. These factors suggest that the chemical inhibition of OASS isoforms could be a promising strategy for the development of antibiotic adjuvants [4].

Heterocyclic compounds have been reported over the years to have numerous biological activities and many biologically relevant compounds including phytochemicals and secondary metabolites contains an heterocyclic ring in their structure [11]. Furthermore, the conventional method for the discovery of bioactive molecules has been reported to be expensive and time-consuming due to the low success rate observed, especially at the later stage of the development process. Virtual screening is one of the strategies that is used to optimize the drug discovery process [12]. Advances in bioinformatics and computational modeling are some of the drivers of modern drug discovery that have enabled the virtual screening of biologically active compounds in the identification of hits and lead compounds [13]. The latest approach to discovering antibacterial drugs is represented by recent developments in computer-aided drug design (CADD), parallel and high-performance computing (HPC) platforms, and innovative in silico approaches [14]. The accuracy of high-throughput virtual screening can be improved using machine learning methods via ligand-based, structure-based, or consensus-based approaches [15]. Hence, this study aims to identify potential inhibitors of *St*OASS that have better inhibitory potential than co-crystallized ligand PLP using the CADD approach, and then, optimize these compounds to design novel inhibitors with good synthetic accessibility scores and improved pharmacodynamics and pharmacokinetics profiles that can serve as antimicrobial adjuvants.

## 2. Results

### 2.1. Pharmacophore Modeling

**Figure 1:**
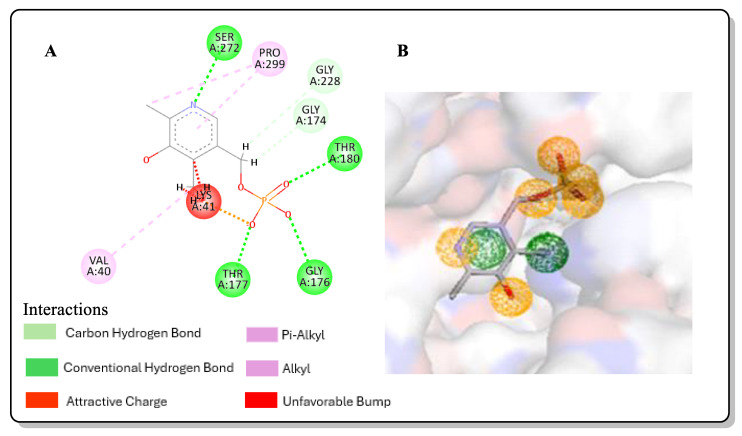
(**A**) 2D interaction of PLP and 1OAS; (**B**) representative features in Pharmit.

The pharmacophores that are responsible for the interaction present in co-crystallized ligands are the pyridine ring and the phosphate group (Figure 1), as viewed in Discovery studio. These interactions and the features that they correspond to on the Pharmit interface are as follows: the pyridine ring-and-*St*OASS interaction corresponds to hydrogen bond donor and aromatic ring features, and the phosphate group interaction corresponds to four hydrogen bond donor features. These features conferred the resulting binding affinity of pyridoxal 5-phosphate (PLP) and *St*OASS; hence, they were key to searching for pharmacophores within the PubChem database that had similar features, also taking how features are positioned into consideration.

### 2.2. Molecular Docking

A total of 4255 compounds, including PLP, were docked into the active site of *St*OASS, and the best 10 hits were recorded. The top 10 hits from the virtual screening had binding energies ranging from −9.1 to −8.5 kcal/mol, which were all higher than the binding energy of the cofactor PLP at −5.7 kcal/mol. The higher binding affinity of these compounds compared to PLP suggests that they have the likelihood of competitively binding to the active site of *St*OASS, thereby inhibiting it, making them possible antibacterial adjuvants. The docking scores of the best ten hits are presented in Table 1.

### 2.3. Post-Screening Analysis

**Figure 2:**
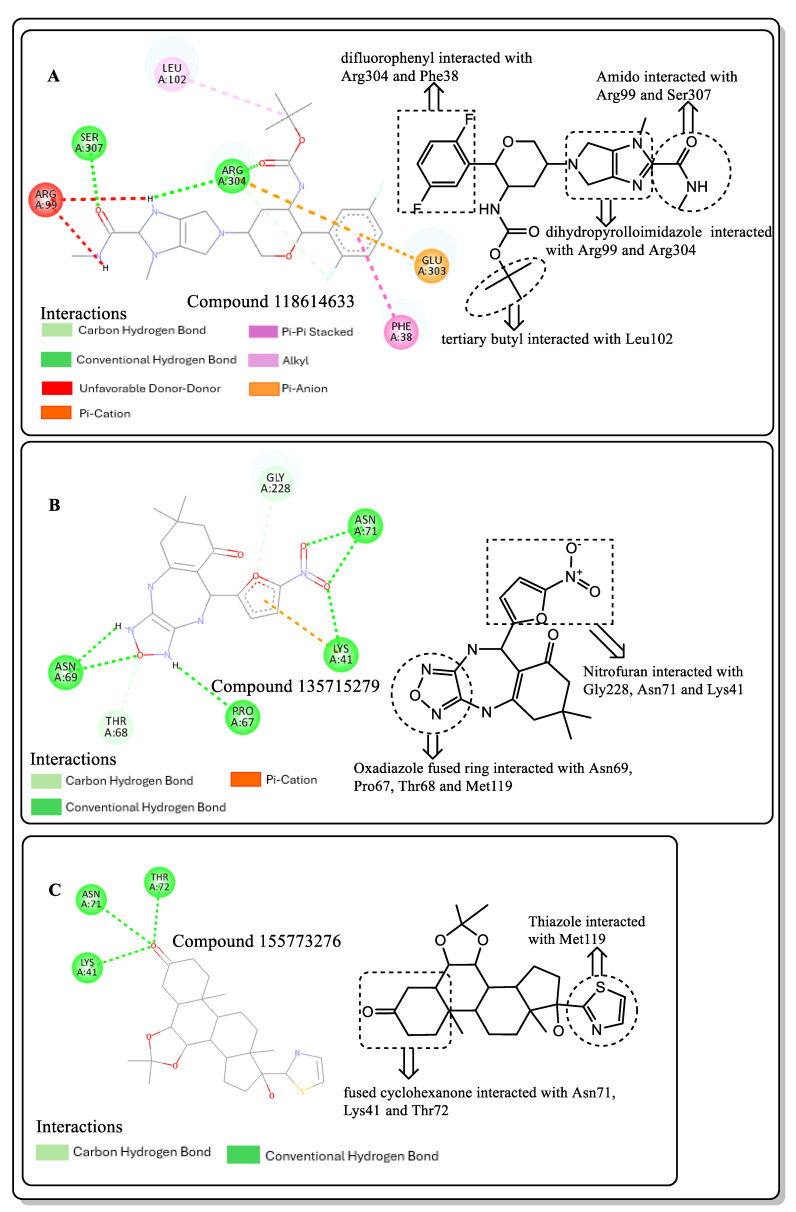
Two-dimensional interaction of best hits: (**A**) compound 118614633, (**B**) compound 135715279, and (**C**) compound 155773276 in active site residue of *St*OASS showing moieties involved in the interaction.

Various interactions between the atoms of the best hits with the amino acid residues in the active site of *St*OASS were viewed and analyzed using the Discovery Studio 2021 client. The intermolecular hydrogen bonds that formed between the ligand and the amino acids in the active sites improve the strength of the protein–ligand complex, emphasizing the importance of hydrogen bond acceptors (HBAs) and donors (HBDs) in the ligand’s structural design [16]. Post-screening analyses revealed interactions such as conventional hydrogen bonds, carbon–hydrogen bonds, and pi–cation, pi–anion, pi-pi stacking, alkyl, and pi–alkyl bonds. The binding affinities and docking scores obtained for each of the best hits were considerably influenced by interactions. Compound 118614633, the best hit from the screening with a binding energy of −9.1 kcal/mol, has nine HBAs and two HBDs, which were responsible for the three hydrogen bond formations at the amino acid residues at the *St*OASS active site (Figure 2). The hydrogen bonds were formed with Ser307 and Arg304 residues. Other interactions include pi-pi stacking with Leu102 and Phe38, and pi–anion interaction with Glu303. Compound 135715279, with a binding energy of −8.9 kcal/mol, was the second best amongst this series of compounds. The interactions with the amino acid residues at the *St*OASS active site included five hydrogen bond interactions with Asn69, Pro67, Asn71, Lys41, and Gly228, and Pi–Carbon and Pi–Alkyl interactions with Met119 and Lys41, respectively (Figure 2). Compound 155773276 showed the third best binding affinity, with three HBAs and two HBDs making three hydrogen bonds with Lys41, Asn71, and Thr72 (Figure 2). The strength of the complex produced is significantly increased by the intermolecular hydrogen bonds formed between the ligand and amino acids in the active sites, and this improves the docking scores [17]. Therefore, Lipinski’s rule of five, which states that a drug candidate should contain HBA ≤ 10 and HBD ≤ 5, emphasizes the importance of hydrogen bond acceptors (HBAs) and donors (HBDs) in the ligand structure.

### 2.4. Structural Activity Relationship (SAR) of Best Hits from Docking Study

The SAR of the best three hits was examined to highlight the scaffolds and functional groups that were involved in its interaction with *St*OASS, and hence, its corresponding binding affinity, as these were considered in the optimization of the hit compounds to lead compounds. Compound 118614633, with the highest binding affinity, has a dihydropyrolloimidazole heterocycle with an amido group linked directly to it. The amido carbonyl interacted with the *St*OASS active site residue Arg99 via hydrogen bonding and amido NH with Ser307 via an unfavorable donor–donor interaction. Dihydropyrollo N interacted with Arg99 and Arg304 via unfavorable donor–donor and conventional hydrogen bonds, respectively. Another scaffold present in this compound is difluorophenyl, with the phenyl ring interacting with Arg304, Glu303, and Phe38 via two Pi–cation and one alkyl interaction. The central carbon of the tertiary butyl substituent introduced an alkyl interaction with Leu102. Compound 135715279 has a diazepine-fused oxadiazole linked to a nitrofuran. The oxadiazole-fused heterocycle interactions were as follows: NH at position one interacted with Pro67 via conventional hydrogen bonding; O at position 2 interacted with Thr68, Met119, and Asn69 via a carbon–hydrogen bond, a pi–alkyl interaction, and a conventional hydrogen bond, respectively; and NH at position 3 interacted with Asn69 via conventional hydrogen bonding. The nitro group of the nitro furan interacted with Asn71 and Lys41 via a conventional hydrogen bond, the furan O interacted with Gly228 via a carbon–hydrogen bond, and the Pi system of the furan ring interacted with Lys41 via carbon–hydrogen bonding. Compound 155773276 has a thiazole ring as its major heterocyclic compound, which formed a carbon–hydrogen bond and a Pi–alkyl interaction with Met119. Another moiety of interest within this compound is the cyclohexanone fused ring, whose carbonyl interacted with Lys41, Thr72, and Asn71 via conventional hydrogen bonding.

### 2.5. In Silico Toxicity and Druglikeness Prediction

The pharmacokinetic characteristics and toxicity hazards of all the top hits as predicted by OSIRIS Property Explorer are shown in Table 2. The standard of 500 g/mol was established because substances with lower MWs tend to be distributed more easily than those with greater MWs [18]. The MWs of the best hits were between 341.0 and 437.5 g/mol, which is within the acceptable range. The logarithm of the partition coefficient between n-octanol and water yielded the clog P value. Values below 5.0 are acceptable; however, values above 5.0 denote low hydrophilicity or poor absorption [19]. The best 10 hits had clog P values below 5.0, indicating that all the compounds have good absorption capacity. A TPSA score less than 160 Å^2^ is regarded acceptable, indicating that the compounds will have good oral bioavailability [20], all 10 best hits’ TPSA scores were acceptable. Solubility (log S) influences both absorption and distribution; values more than −4 are regarded acceptable, as this corresponds to a score of more than 80% of marketed drugs. The best three hits all had logS values greater than −4. The higher the drug score value, the higher the compound’s chance of being considered a drug candidate. Compound 123531073 had the highest drug score with a value of 0.56. The toxicity properties of the three best hits, 118614633, 135715279, and 155773276, suggest mild toxicity tendencies of these compounds, implying their predictive drug conformity, compatibility, and safety *in vivo*.

### 2.6. Lead Optimisation via Scaffold Hopping

**Figure 3:**
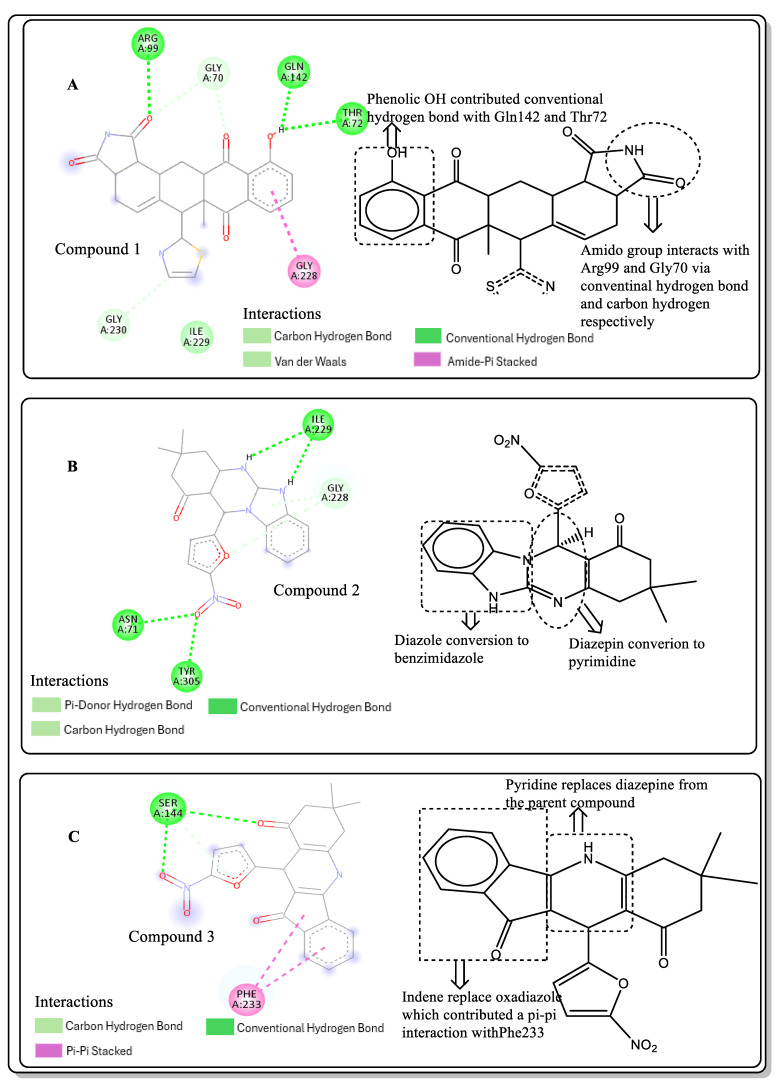
Binding interactions of optimized best hits: (**A**) lead compound **1,** (**B**) lead compound **2,** and (**C**) lead compound **3** at active site of *St*OASS.

Further optimisation of the best three compounds, 118614633, 135715279, and 155773276, was performed using ADMETopt [21], which optimizes via scaffold hopping. This generated a total of 189 compounds which were docked against *St*OASS. Lead compound **1** (Figure 3), with a binding energy of −9.3 kcal/mol, has similarity to compound 155773276 as its parent compound. The thiazole moiety was maintained, and the other component was substituted, having an amido group and a phenol group. The amido carbonyl interacted with the *St*OASS active site residues Arg99 and Gly70 via a carbon–hydrogen bond and conventional hydrogen bond. The phenolic OH interacted with Gln142 and Thr72 via conventional hydrogen bonding. The presence of these groups can be said to have contributed a synergistic effect as there was a noticeable increase in binding affinity when the two compounds were compared. Lead compounds **2** and **3** had binding energies of −9.1 kcal/mol and −8.8 kcal/mol, respectively, having similarities with compound 135715279 as their parent compound, with a binding energy of −8.9 kcal/mol. The scaffold that was constant for all three compounds is nitrofuran. Lead compound **2** (Figure 3) had its diazepine-fused oxadiazole substituted with an imidazolopyrimidine-based moiety, which interacted via conventional hydrogen bonding with Ile229, and the imidazole pi system interacted with Gly228 via pi–donor hydrogen bonding. These interactions introduced a synergistic effect, and hence, an increase in the binding affinity of the compound. Lead compound **3** (Figure 3) had its diazepine-fused oxadiazole substituted with an indene-fused quinolinone. The indene pi system interacted with Phe233 via pi-pi stacking, and the carbonyl of the quinolinone interacted with Ser144 via conventional hydrogen bonding. These interactions introduced an antagonistic effect as the binding affinity of this compound was reduced when compared to the parent compound.

#### Druglikeness and Toxicity Profiling of Optimized Compounds

The medicinal chemistry scores of the best three hits amongst the optimized compound and toxicity profiles were compared to their parent structures, shown in Table 3. ADMETlab was used in toxicity and druglikeness profile prediction [22]. The results showed that the optimized compounds as compared to their parent compound were not blood–brain barrier permeants, and the AMES toxicity, which is an indication of mutagenic potential [23], was reduced for lead compound **1** compared to its parent compound 155773276 and remained the same for lead compounds **2** and **3** when compared with their parent compound 135715279. The quantitative estimated druglikeness (QED) scores of the optimized best hits were predicted to be >0.34, with all values within the range of 0.51–0.62, indicating that their structures are not too complex based on the concept of desirability [24]. The synthetic accessibility score, which is a measure of the ease of synthesis of the compounds, was <6 for all three compounds, indicating that optimized compounds are easy to synthesize [25], with lead compound **3** having a score of 0.35. The medicinal chemistry evaluation (MCE-18) scores, where MCE-18 ≥ 45 is a suitable value, were 121.97, 95.92, and 98.57 for 118614633, 135715279, and 155773276, respectively.

### 2.7. Molecular Dynamics Simulation

#### 2.7.1. Root Mean Square Deviation and Root Mean Square Fluctuation

**Figure 4:**
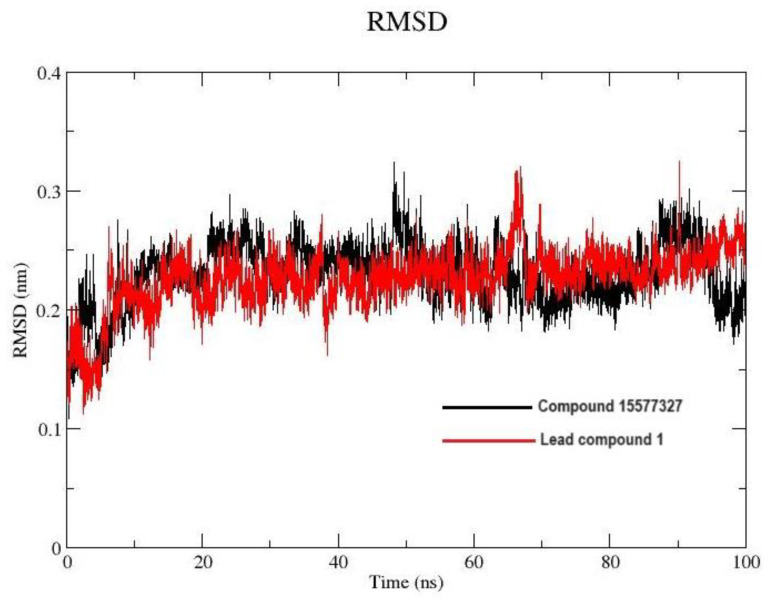
Protein Cα-RMSD plot in complex with compound 15577327 and lead compound **1**.

The Root Mean Square Deviation (RMSD) and Root Mean Square Fluctuation (RMSF) of the MD trajectories were used to comprehend the stability of and possible fluctuations in the complexes [26]. The RMSF is used for quantifying local changes/amino acid fluctuations along the protein chain, with less RMSF fluctuation indicating less flexibility. The RMSD of the c-alpha protein backbone fluctuated between 1.5 and 3 Å, with an average RMSD of 2.67 Å for lead compound **1** and 2.33 Å for its parent compound 155773276 when bound to the active site of protein. This suggests that target conformation was relatively stable during the simulation even when bound to ligands; this is depicted in a plot of RMSD against frame number (Figure 4).

**Figure 5:**
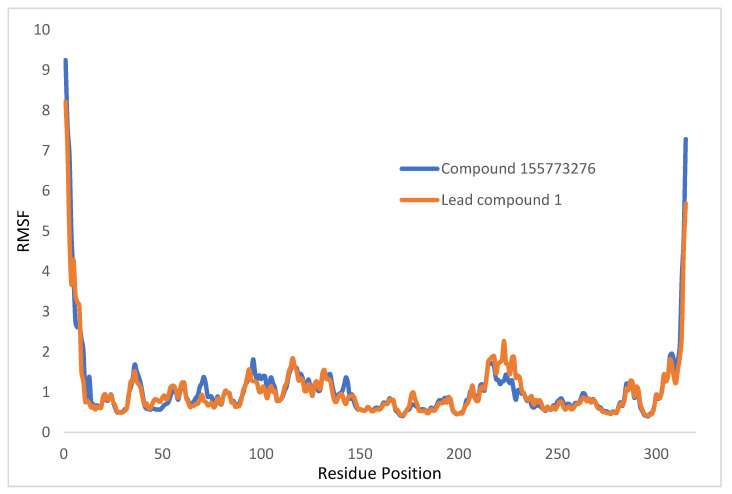
Protein Cα-RMSF plot in complex with compound 155773276 and lead compound **1**.

A plot of RMSF against the residue position of the c-alpha protein backbone is shown in Figure 5, with the fluctuation ranging between 0 and 2 Å. When lead compound **1** and compound 155773276 were present at the active site of target, the range was lower for compound 155773276, indicating that it forms a more stable complex with target.

#### 2.7.2. Principal Component Analysis

**Figure 6:**
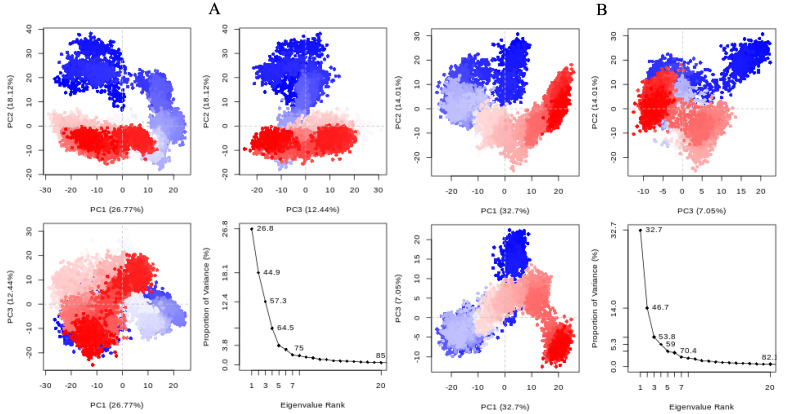
PCA plots and eigenvalue rank plots of the protein backbone in the complex with (**A**) Compound 155773276 and (**B**) lead compound **1**.

*St*OASS protein atoms are converted to a group of uncorrelated principal components (PC), with a plot showing how these components are correlated (Figure 6) [12]. PC1 for compound 155773276 accounted for 26.77% of the cumulative variance, while PC2 and PC3 were responsible for 18.12% and 12.44%, respectively, giving a total of 57.3% for the first three PCs, as shown in the eigenvalue rank plot. The total of the first three PCs for lead compound **1** was 53.8% of the cumulative variance, with PC1 alone accounting for 32.7%. This suggests that the molecular dynamics simulation captured the major or dominant motions rather than the less dominant ones, considering the first three PCs were greater than 50% of the total principal components for both complex systems.

#### 2.7.3. Radius of Gyration

**Figure 7:**
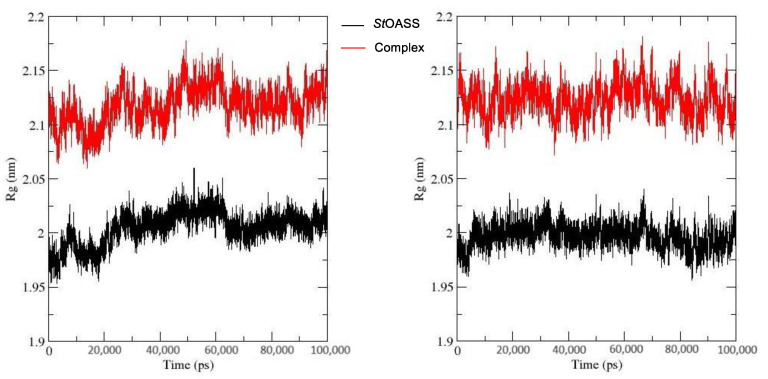
Rg plot of *St*OASS (black) and (**A**) *St*OASS_compound 155773276 (Red) (**B**) *St*OASS_lead compound **1** (Red).

The radius of gyration (Rg) is a parameter used to indicate the total size of a chain molecule [27]. It can be used to quantify the degree of structural variation in proteins during molecular dynamics simulations. It assesses the protein’s flexibility and compactness within a biological context, comparing the protein’s structure over time to the hydrodynamic radius that may be observed through experimentation [28]. The *St*OASS_lead compound **1** complex had a higher radius of gyration and the values were relatively stable throughout the simulation run. This implies that the *St*OASS_lead compound **1** complex had more flexibility/lower rigidity, compared to the *St*OASS_compound 155773276 complex, which had observable variation in its Rg value throughout the simulation run (Figure 7).

#### 2.7.4. Solvent-Accessible Surface Area

**Figure 8:**
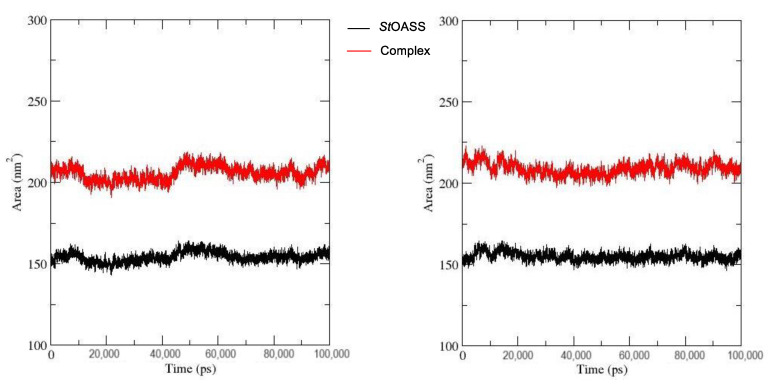
SASA plot of *St*OASS (Black) with (**A**) *St*OASS_compound 155773276 (red) and (**B**) *St*OASS_ lead compound **1** (red).

The percentage of a biomolecule’s surface that interacts with water is measured by the Solvent-Accessible Surface Area (SASA) [29]. An high SASA value indicates protein exposure to the surrounding solvent surface area, whereas a low SASA value indicates less exposure to the surrounding solvent and hence more stability [26]. The SASA calculation of *St*OASS_lead compound **1** complex decreased at around 10 ns and was relatively stable to the end of the simulation run (Figure 8). This indicates a stable conformation and that the complex was least exposed to the solvent as compared to its parent *St*OASS_compound 155773276 complex, where a decrease in SASA was first observed around 10ns followed by an increase in SASA observed at around 50 ns.

#### 2.7.5. Hydrogen Bond Analysis

**Figure 9:**
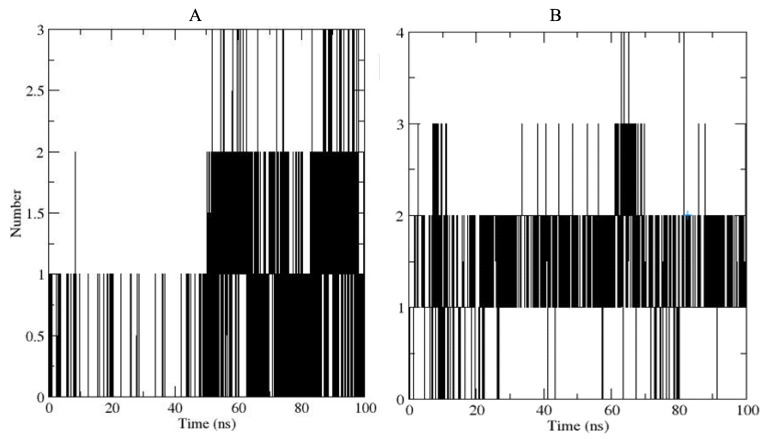
Hydrogen bonds formed between (**A**) compound 155773276 and (**B**) lead compound **1** at the active site of *St*OASS during the production run.

H-bond formations are important in the stability of complexes during MD simulation [30]. Multiple hydrogen bonds were identified between the active site of the protein and the ligand; these are shown in Figure 9. A total of four H-bonds were observed during the MD simulation of lead compound **1** at the active site of *St*OASS, and four H-bonds were observed for compound 155773276. This corresponded with the observed H-bond interaction in the molecular docking study either through conventional hydrogen bonds, carbon–hydrogen bonds, or van der Waals interactions between the ligand and protein.

## 3. Discussion

An effective way to combat AMR may be to utilize antibiotic adjuvants. Bacterial metabolic pathways, such as the biosynthesis of cysteine, may be of particular importance and were employed in this study. The enzyme being considered is the *O*-acetyl serine sulfhydrylase of *Salmonella typhimurium*, which catalyzes the final step of cysteine biosynthesis, implying high selectivity and safety of its specific inhibitors [31]. The 10 best hits have higher binding affinities than the known co-ligand, pyridoxal 5’-phosphate, according to the docking scores from the virtual screening of compounds from PubChem databases against the *St*OASS. The binding interaction and moieties inducing the interactions were identified via post-screening analysis of the best three hits (Table 1). Compound 118614633 had the lowest binding energy of −9.1 kcal/mol, and hence, the highest binding affinity. The pharmacophore features of a compound are a determinant of the activity of the compound against a target. The moieties and functional groups involved in the interaction of compound 118614633 and the active site of *St*OASS were difluorophenyl, which corresponds to an aromatic and hydrophobic feature; the dihydropyrolloimidazole hybrid, which corresponds to one of the two HBD and two of the nine HBA features; tertiary carbon, which corresponds to the hydrophobic feature; and the amido group, which corresponds to hydrophobic and hydrogen donor features (Figure 2). Compound 135715279 had the next best binding affinity, with a docking score of −8.9 kcal/mol, with moieties that were involved in the interaction being the nitrofuran scaffold, which corresponds to aromatic features, two hydrogen acceptors, and hydrophobic features, and the oxadiazole moiety, which corresponds to two hydrogen donors and one hydrogen acceptor. The HBA and HBD features of ligands provided a point of hydrogen bond interaction that formed between ligands and the target (Figure 2). Compound 155773276, with a docking score of −8.8 Kcal/mol, had two parts of its structure responsible for the interaction with the active site of *St*OASS; they are the thiazole moiety, which corresponds to the hydrogen donor and hydrophobic features, and the cyclohexane, which corresponds to the hydrogen acceptor feature (Figure 2). These features also play a role in determining the pharmacodynamic and pharmacokinetic properties of a compound; an example is hydrophobicity, which determines the distribution of compounds in the cell membrane [32]. Hit-to-lead optimization via scaffold hopping was carried out on the best three hits, giving rise to compounds **1**, **2**, and **3**. Compounds **1** and **2** had an increased binding affinity when compared with their parent compounds. The toxicity profile of optimized compounds was also compared with their parent compounds. Lead compound **1** showed an improvement in its profile when compared to its parent compound, 155773276, for AMES toxicity, implying that it has lower mutagenic potential and BBB permeation probability (Table 3). The generated trajectories from the MD simulation were used to analyze the behavior of each complex in the explicit water environment. The RMSD plot of lead compound **1** and 155773276 at the target active site suggests that the ligands are stable, as the average RMSD for each complex was below 3 Å (Figure 4). The RMSF plot showed the fluctuations of each ligand at the active site of the protein, with similar fluctuation patterns during the MD simulation process (Figure 5). The eigenvalue rank plot (Figure 6) shows the variance proportion resulting from each principal component (PC), with the first three PCs (PC1, PC2, and PC3) of each complex accounting for more than 50% of its total variance for both complexes. The radius of gyration (Rg) showed the compactness of the complex, which in both complexes was higher in value than the unbound protein (Figure 7), and the SASA values of both complexes (Figure 8) were higher than the unbound protein, indicating that the presence of the ligand at the active site of *St*OASS increases its exposure to the surrounding solvent. The hydrogen bond analyses (Figure 9) showed that the ligands maintained stable conformation at the active site of the protein during the simulation, which corresponded to the hydrogen bonds observed during the molecular docking studies, suggesting the potential of these ligands as inhibitors of *St*OASS.

## 4. Materials and Methods

### 4.1. Protein Structure Preparation

The crystal structure of *St*OASS (PDB ID: 1OAS) was collected from the Protein Data Bank. Co-ligand pyridoxal 5 phosphate (PLP) was separated from the protein. The polar hydrogen atoms and Kollman charges were added to the protein, and the water molecules were eliminated; this was then minimized using UCSF Chimera software v 1.14 and saved in PDB format for further analysis.

### 4.2. Pharmacophore Modeling

A ligand-based pharmacophore model based on the interaction of the crystalized 3D structure of *St*OASS (PDB ID; 1OAS) and its co-crystallized ligand, pyridoxal 5-phosphate (PLP), was carried out using the Pharmit server [33]. The key features used to construct an effective pharmacophore query include hydrogen bond acceptors, hydrogen bond donors, hydrophobicity, and aromaticity. These features were chosen based on the 2D interaction of PLP and the binding site of 1OAS (Figure 1). The hit screening parameters were also set to obey Lipinski’s rule of five [34]. These set parameters were used to search through the updated PubChem database, returning a total of 5340 compounds.

### 4.3. Ligand Library Preparation

The “sdf” file formats of the 5340 compounds obtained from the pharmacophore-based screening were downloaded, and the OpenBabel panel [35] of the PyRx-0.8 software [36] was used to view these compounds and convert them to their corresponding 3D structures. The successful conversion of 4254 compounds to the docking format “pdbqt” as opposed to all 5340 was because of the inability to set up a force field for the remaining compounds. Pyridoxal 5-phosphate was also added to the ligand library of 4254 compounds for the docking simulation; this is because the aim was to develop compounds that have higher binding affinity compared to the co-ligand of *St*OASS and would readily bind to its active site, thereby inhibiting the activity of the enzyme [37].

### 4.4. Virtual Screening and Post-Screening Analyses

Autodock vina was used to carry out the molecular docking studies [38]. Firstly, the protein was converted to “pdbqt” format and a grid box was set to cover the active site of the crystal structure with the following dimensions in Å: center (X, Y, Z) = (13.42, 7.19, 35.65); dimensions (X, Y, Z) = (5.72, 6.39, 14.01) with an exhaustiveness of 8. The Discovery Studio 2021 Client was used in post-docking analysis [39]. An extensive qualitative structural assessment and examination of the structural representations of the three best hits were carried out to examine the scaffolds and functional groups responsible for the binding interactions observed in the post-screening analysis.

### 4.5. Hit-to-Lead: Best Hit Optimization

The best 3 hit compounds were optimized using Admetopt to achieve structures that are novel with good synthetic accessibility scores and improved pharmacodynamics and pharmacokinetics profiles. The scaffolds and features responsible for interactions in the best 3 hits were taken into consideration in building the optimized lead compounds.

### 4.6. In Silico Druglikeness Prediction

The failure of drug candidates at the testing or clinical trial stage or their call back after they have entered the market due to toxicity, poor pharmacodynamic or pharmacokinetic properties, or reported side effects, amongst others, are problems that come to play during the drug development process [40]. Hence, a predictive ADMET study was conducted using the OSIRIS Property Explorer tool [41] to estimate the toxicity profile and druglikeness of the best 10 hits to propose whether they can pass as being orally active drugs [42]. The following pharmacokinetic properties were examined: molecular weight and solubility (log S), which are determinants of the degree to which a compound can penetrate the biological membrane [39]; hydrophilicity (log P) to estimate the dissolution of compounds in a liquid membrane; topological polar surface area (TPSA) associated with membrane permeability [43]; and drug score, a single value from the combination of all the properties listed above [44] and toxicity risk combining irritant, tumorigenic, mutagenic, and reproductive risks [45]. Druglikeness, a value that determines the consistency in properties of a compound as a drug candidate, was also examined [46]. The higher the drug score value, the higher the chance of the compound being considered a drug candidate [44].

### 4.7. Molecular Dynamics Simulation

Complexes of the target and best hit from the docking study, and best optimized compound, were subjected to molecular dynamics simulation (MDS) using GROMACS in Ubuntu 20.04.6. This was carried out to determine the stability of ligand(s) at the active site of the target [42]. The ligand topology was generated using the Swiss Param web server [47] and target topology generated using the CHARMM27 forcefield. The ligand and target were merged to form the complex; then, triclinic water boxes with a distance of 1nm and a transferable intermolecular potential (TIP) 3-point water model were deployed for the solvation of the complexes. Energy minimization of 10,000 steps was performed, and a production simulation run was carried out for 50,000,000 steps (100 ns) at a temperature of 300 K as defined in the script alongside other parameters [48]. Analysis of the simulation results was performed to determine the Root Mean Square Deviation (RMSD), Root Mean Square Fluctuation (RMSF), radius of gyration (RoG), Solvent-Accessible Surface Area (SASA), and hydrogen bond (h-bond) analysis. The Galaxy Europe platform Bio3D tool was then used to perform Principal Component Analysis (PCA).

## 5. Conclusions

In this study, a ligand library was generated from the PubMed database based on the interactions of co-ligand PLP at the active site of *St*OASS. These ligands were docked against the same target. The best 10 hits were recorded and were in the order 118614633 > 135715279 > 118505490 > 123531073 > 155773276 > 132083481 > 153409783 > 136030136 > 153368440 > 156238864, and all performed favorably when compared to the binding energy of PLP. The best three hits were then optimized, giving lead compounds **1**, **2**, and **3**, with lead compound **1** being the best-optimized compound based on binding affinity, druglikeness, and toxicity profile, performing better than its parent compound. These compounds were also predicted to be stable at the active site of the target based on MD simulation result analysis. These results infer that all 10 best hits and lead compound **1** could be possible specific inhibitors of StOASS, and hence, possible adjuvants that can be used in combination with antimicrobial agents, as they possess higher inhibitory and drug-like properties than the cofactor pyridoxal 5’-phosphate. This combination therapy might also pave the way for solving the drug resistance issue in old existing antimicrobial agents.

## Figures and Tables

**Table 1:** Binding affinity of 10 best hits from molecular docking of compounds and *St*OASS.

PubChem ID	3D Structure	Docking Score (kcal/mol)	nHBA	nHBD
118614633	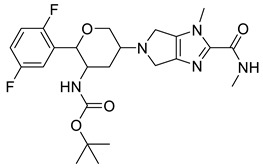	−9.1	9	2
135715279	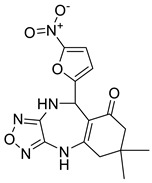	−8.9	10	2
155773276	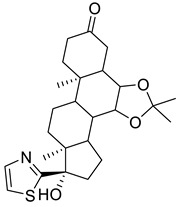	−8.8	5	1
118505490	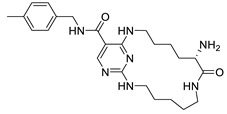	−8.8	9	5
123531073	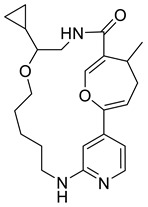	−8.8	6	2
132083481	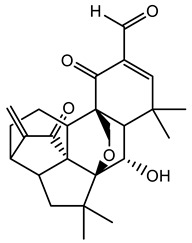	−8.8	5	1
153409783	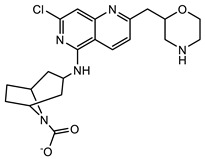	−8.6	8	2
136030136	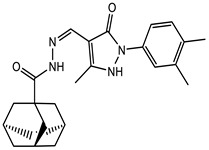	−8.5	6	2
153368440	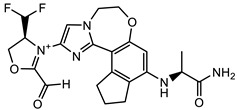	−8.5	9	3
156238864	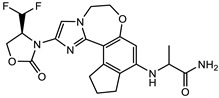	−8.5	9	3
Plp	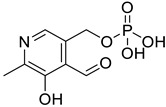	−5.6	7	3
Gentamicin	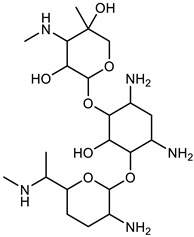	−7.8	12	11

nHBA: number of hydrogen bond acceptors; nHBD: number of hydrogen bond donors.

**Table 2:** Druglikeness and toxicity profiles of best hits using OSIRIS Property Explorer.

PubChem ID	MW (g/mol)	Clogp	Tpsa (Å^2^)	logS	Drug Score	Mutagen	Tumorigenic	Irritant	Reproductive Effective
118614633	491	2.29	102.5	−3.04	0.36	Low	Low	Low	Low
135715279	345	1.27	139.3	−3.49	0.43	Low	Low	Low	Low
118505490	453	1.99	134.0	−3.73	0.32	Low	Low	Low	Low
123531073	397	2.37	72.48	−3.60	0.57	Low	Low	Low	Low
155773276	445	3.48	96.89	−4.37	0.30	Low	Low	Low	High
153409783	431	−0.59	99.08	−1.93	0.27	Medium	High	Low	Medium
136030136	406	2.62	73.8	−6.18	0.35	Low	High	Low	Low
153368440	461	1.23	111.7	−5.08	0.19	High	Low	Low	Low
156238864	447	1.9	111.7	−5.40	0.29	Low	Low	low	Low
PLP	247	−3.2	126.7	−1.20	0.29	High	Low	low	Low
Gentamicin	477	−4.21	199.7	−0.59	0.77	Low	Low	low	Low

**Table 3:** Druglikeness of optimized compounds compared with their parent compound.

Compound ID	Medicinal Chemistry Score					Toxicity Profile			
	QED	Synthetic Accessibility	MCE	GSK	Pfizer	HHT	AMES	hERG Blocker	BBB
PubChem 155773276 Compound **1**	0.68	4.92	142.09	Rejected	Rejected	-		---	+++
	0.51	4.56	121.97	Rejected	Accepted	--	--	-	--
PubChem 135715279 Compound **2** Compound **3**	0.62	3.95	81.14	Accepted	Accepted	+	+++	---	+++
	0.62	3.49	95.92	Accepted	Accepted	++	+++	---	--
	0.61	0.35	98.57	Accepted	Accepted	-	+++	---	---

Prediction probability value range: 0–0.1 (---), 0.1–0.3 (--), 0.3–0.5 (-), 0.5–0.7 (+), 0.7–0.9 (++), 0.9–1.0 (+++).

## Data Availability

The original contributions presented in this study are included in this article. Further inquiries can be directed to the corresponding author.
